# Adults with mild to moderate depression exhibit more alcohol related problems compared to the general adult population: a cross sectional study

**DOI:** 10.1186/s12889-015-1837-8

**Published:** 2015-06-09

**Authors:** Julia Åhlin, Mats Hallgren, Agneta Öjehagen, Håkan Källmén, Yvonne Forsell

**Affiliations:** Department of Public Health Sciences, Section for Epidemiology and Public Health Intervention Research (EPHIR), Karolinska Institutet, Stockholm, Sweden; Department of Clinical Sciences, Psychiatry, Lund University, Lund, Sweden; Department of Clinical Neuroscience, Karolinska Institutet, Stockholm, Sweden; STAD, Centre for Psychiatry research, Stockholm, Sweden

## Abstract

**Background:**

Alcohol use has been shown to interfere with treatment for depression, but consumption habits are not routinely screened in primary care. To date, few studies have compared the alcohol consumption habits of patients with depression to the general population. The purpose of this study was to compare alcohol habits in adults diagnosed with depression in primary care to the general adult population in Sweden.

**Methods:**

Nine hundred fourty six patients diagnosed with mild to moderate depression, without a primary substance use disorder, in primary care settings located across Sweden completed the Alcohol Use Disorders Identification Test (AUDIT). Consumptions habits and alcohol related problems in the depressed sample were compared to those in the general adult population (n = 663). Analyses were stratified by gender and age.

**Results:**

Ratings of alcohol problems and measures of hazardous drinking and binge drinking were significantly higher among patients seeking treatment for depression in primary care compared to the general population. Male patients scored higher on the AUDIT total and AUDIT-C (consumption) subscale than men in the general population. Compared to younger adults (aged 17–27) older depressed adults (aged 28–50 and 51–71) exhibited higher rates of consumption and problems related to alcohol.

**Conclusions:**

Compared to the general adult population, consumption and problems related to alcohol use were substantially higher among patients with mild to moderate depression in primary care. Routine screening of alcohol use in primary care is recommended for patients presenting with depression.

## Background

Alcohol use disorders frequently co-exist with mood disorders [1], yet little is known about the alcohol habits of patients seeking treatment for depression in primary care [2]. In Sweden, national guidelines recommend screening for alcohol problems within health care services [3, 4]. Clinicians are increasingly trained in screening and brief intervention (SBI) techniques; however, routine screening of hazardous drinking habits and alcohol use disorders is still not an entrenched part of clinical practice [5]. This is potentially problematic because alcohol use, even at ‘moderate’ levels, can have an adverse impact on treatment outcomes for depression [6–8]. To date, research examining the prevalence of alcohol problems in depression has focussed mainly on patients with alcohol dependence in psychiatric inpatient clinics [2]. Most people with depression initially seek help within the primary care sector as outpatients, which is a recommended first step. More information is needed about alcohol habits in patients with less severe forms of depression, including the prevalence of hazardous or risky drinking [9]. The present study addresses this research gap by assessing patterns of alcohol use and problems related to alcohol in a sample of Swedish adults seeking treatment for mild to moderate depression in primary care. Results are compared to consumption habits in the general adult population. Implications for screening and assessment are discussed.

## Methods

### Participant recruitment

Data from two population surveys were analysed. Patients in the depressed sample were recruited via primary care centres located in six county councils in Sweden; a total population of approximately 6 million people. All patients in the depressed sample were involved in a randomized controlled trial (RCT) - ‘Regassa’. The project aims to study the effects of internet based cognitive behavioural therapy (ICBT), physical activity and treatment as usual on sick-leave and work-ability in patients with mild to moderate depression, anxiety and stress related mental ill-health. Patients aged 18 years and above who scored >9 on the Patient Health Questionnaire (PHQ-9) [10] were invited to participate. Recruitment began in February 2011 and the last patient was assessed in January 2013. Exclusion criteria were age (<18 years), non-Swedish speaking (i.e. not being able to read and understand the questionnaires and material), having a severe somatic illness, a *primary* substance use disorder or a psychiatric diagnosis that required specialist treatment (e.g. psychosis). Written informed consent was obtained from the patients before they were randomized to one of the three treatment conditions. Following an initial consultation with their primary health care provider, eligible patients who agreed to participate completed a baseline questionnaire administered by a research nurse. The questionnaire included a demographic survey, the Alcohol Use Disorders Identification Test (AUDIT – described below), and the Montgomery-Åsberg Depression Rating Scale (MADRS) [11]. A psychiatric diagnostic assessment (MINI International Neuropsychiatric Assessment) [12] was performed to confirm the diagnosis. In total, 946 patients met the inclusion criteria for the trial, agreed to participate and were included in the analyses. A flow-chart of the participants’ progress through the trial is presented elsewhere [13]. The Regional Ethical Review Board at the Karolinska Institute approved the study (Dnr 2010/1779-31/4).

Data from the depressed sample was compared to AUDIT data from a general population survey conducted in 2009. Data from the latest study of alcohol habits in the general Swedish population showed that the changes between 2009 and 2014 were insignificant [14]. The sampling methodology has been described previously [15]. Briefly, a random sample of 1250 persons was drawn from a national register (Dafa/Spar). The potential respondents were aged 17–71 with a Swedish address. Respondents received a paper questionnaire with the AUDIT questions and were asked to return the survey by regular mail.

### Materials

Alcohol consumption and problems related to alcohol were assessed with the AUDIT [16]. The instrument has been validated in primary care settings [17] and is suitable for use in the general population. The Swedish version of AUDIT has shown acceptable psychometric properties [18]. Here, we report the total AUDIT score, the consumption subscale (AUDIT-C) items 1–3, and the alcohol problems subscale (signs of dependency and harms combined, items 4–10). Studies have shown that a two-factor AUDIT scale consisting of consumption and alcohol problems is more valid than the original three-factor scale consisting of consumption, dependence and harms [18, 19]. The proportion of ‘hazardous drinkers’ [17] was based on the total score ≥8 for men [16] and ≥6 for women [20]. The cut-off ≥8 has shown high sensitivity and specificity of AUDIT for hazardous alcohol consumption [16]. The proportion of binge drinkers was measured using item 3 “How often do you have six or more drinks on one occasion?”. The binge frequency cut-off was *at least on a monthly basis* and the same number of drinks indicate binge-drinking for men and women [16]. Alcohol abstention was measured using item 1, “How often do you have a drink containing alcohol?”.

### Statistical analyses

Means and standard deviations for the continuous variables were calculated and compared using independent *t*-test and Analysis of Covariance (ANCOVA). ANCOVA was used to adjust for the uneven gender distributions in the general population and in the clinical sample when analysing the continuous variables. Chi-square tests were used to compare the proportion of hazardous drinkers, binge drinkers and abstainers in the depressed and general populations. Analyses were stratified by gender and age (17–27, 28–50 and 51–71 years old), similar to the age distribution used when analysing the general population [15]. Effect sizes (Cohen’s *d*, partial eta squared for continuous outcomes and Odds Ratios for categorical outcomes) were calculated where statistical differences between groups were obtained.

## Results

For the depressed sample, 99.5 % (n = 941) completed a usable AUDIT questionnaire; mean age, 43 years (SD, 12.8; range, 18–71). The majority of participants were female (73 %). In total, 54 % had completed secondary school (year 12); 42 % had completed tertiary education. 78 % of the depressed participants were employed or studying when the assessment was performed. Concurrent depressive and anxiety disorders were most common (74 %), however, part of the sample was exclusively suffering from either depression (9 %) or an anxiety disorder (13 %) as assessed by the MINI. Patients who did not fulfil the MINI criteria for depression nevertheless showed clinically relevant signs of depression as indicated by scores >9 on the PHQ-9, a screening instrument for mood disorders [10]. These individuals were included in the analyses. The mean depression (MADRS) score at baseline was 21.60 (SD, 7.06) indicating that most patients were experiencing a ‘mild to moderate’ depression [21, 22]. In the general population the response rate was 54 % (or 663 persons). The proportion of drop-outs were as follows: men aged 17–27: 55 %, 28–60: 62 %, 61–71: 33 % and women aged 17–27: 48 %, 28–60: 53 %, 61–71: 28 % [15]. Mean age was 46.2 years (SD, 15.8; range, 17–71) and 56 % were female.

### Alcohol consumption and alcohol related problems

AUDIT scores in the depressed sample and the general population stratified by gender (Table 1) and age (Table 2) are shown below. AUDIT total, AUDIT-C and alcohol problems scale were significantly higher in the depressed sample compared to the general population. The proportion of hazardous drinkers and binge drinkers was significantly higher in the depressed sample and the proportion of abstainers was larger in the general population. A gender difference was found; for men, the AUDIT total score, alcohol consumption subscale (AUDIT-C) and the proportion of hazardous drinkers were significantly higher in the depressed sample – an effect not seen among women. Binge drinking was more prevalent among both males and females in the depressed sample. Binge drinking reduced with age, but less in the clinical sample where the proportion of binge drinkers was twice the size as in the general population when comparing the oldest individuals. In the youngest age group there were no significant differences, while among those aged 28–50 and 51–71 the clinical sample had a significantly higher AUDIT total score, alcohol problems score, as well as higher proportions of binge drinking and hazardous drinking. Differences in consumption between the depressed and general population were greatest among the older participants (aged 51–71). Effect sizes for the observed differences between the depressed and general population were small to medium.

**Table 1:** Alcohol consumption habits by gender

	Total n = 1566				Males n = 533				Females n = 1033			
AUDIT variable	General population n = 663	Depressed sample n = 946	Group difference	Effect size	General population n = 276	Depressed sample n = 257	Group difference	Effect size	General population n = 344	Depressed sample n = 689	Group difference	Effect size
AUDIT total mean (SD)	3.99 (3.47)	4.33 (4.15)	F(1,1459) = 10.03**	η_p_^2^ = 0.007	4.73 (4.20)	5.92 (5.05)	t = −2.86**	d = 0.26	3.38 (2.59)	3.74 (3.58)	t = −1.73	-
AUDIT-C mean (SD)	2.76 (2.01)	2.89 (1.94)	F(1,1541) = 7.83**	η_p_^2^ = 0.005	3.16 (2.24)	3.59 (2.17)	t = −2.22*	d = 0.19	2.43 (1.73)	2.63 (1.78)	t = −1.73	-
Alcohol problem scale mean (SD)	0.77 (2.01)	1.45 (2.76)	F(1,1541) = 41.30**	η_p_^2^ = 0.005	1.03 (2.56)	2.35 (3.56)	t = −4.88**	d = 0.46	0.56 (1.38)	1.11 (2.30)	t = −4.72**	d = 0.30
Hazardous drinkers (%)	15	22.4	*χ* ^2^ = 11.66**	OR = 1.63	15.1	28.9	*χ* ^2^ = 13.55**	OR = 2.29	15	20	*χ* ^2^ = 3.41	-
Binge drinker monthly or more (%)	8.1	12.7	*χ* ^2^ = 8.47**	OR = 1.65	13.4	23.3	*χ* ^2^ = 8.83**	OR = 1.97	4.4	8.7	*χ* ^2^ = 6.18*	OR = 2.06
Alcohol abstainers (%)	14.6	10.3	*χ* ^2^ = 6.60*	OR = 0.67	13.1	8.6	*χ* ^2^ = 2.81	-	15.9	11.0	*χ* ^2^ = 5.07*	OR = 0.65

*p < 0.05 **p < 0.01

**Table 2:** Alcohol consumption habits by age group

	17-27 years n = 216				28-50 years n = 783				51-71 years n = 555			
AUDIT variable	General population n = 105	Depressed sample n = 111	Group difference	Effect size	General population n = 243	Depressed sample n = 540	Group difference	Effect size	General population n = 260	Depressed sample n = 295	Group difference	Effect size
AUDIT total mean (SD)	6.13 (5.15)	5.69 (4.98)	F(1,193) = 0.001	-	3.89 (2.77)	4.25 (4.01)	F(1,744) = 6.584**	η_p_^2^ = 0.009	3.16 (2.84)	3.97 (3.95)	F(1,505) = 11.219**	η_p_^2^ = 0.022
AUDIT-C mean (SD)	3.53 (2.60)	3.36 (2.21)	F(1,210) = 0.002	-	2.87 (1.87)	2.87 (1.87)	F(1,768) = 1.862	-	2.36 (1.73)	2.75 (1.93)	F(1,539) = 9.470**	η_p_^2^ = 0.017
Alcohol problem scale mean (SD)	1.77 (3.18)	2.33 (3.35)	F(1,207) = 2.757	-	0.67 (1.46)	1.39 (2.69)	F(1,770) = 23.118**	η_p_^2^ = 0.029	0.43 (1.65)	1.23 (2.57)	F(1,539) = 24.660**	η_p_^2^ = 0.044
Hazardous drinkers (%)	37.9	37.6	*χ* ^2^ = 0.002	-	13.1	21.4	*χ* ^2^ = 6.839**	OR = 1.807	6.8	18.5	*χ* ^2^ = 14.69**	OR = 3.11
Binge drinker monthly or more (%)	21.2	18.2	*χ* ^2^ = 0.299	-	7.0	12.1	*χ* ^2^ = 4.570*	OR = 1.823	5.0	11.6	*χ* ^2^ = 7.79**	OR = 2.50
Alcohol abstainers (%)	16.3	11.8	*χ* ^2^ = 0.909	-	13.2	9.3	*χ* ^2^ = 2.711	-	14.0	11.6	*χ* ^2^ = 0.738	-

*p < 0.05 **p < 0.01

## Discussion

Compared to the general adult population, alcohol consumption and problems related to alcohol use were substantially higher among patients with mild to moderate depression in primary care. The proportion of hazardous drinkers and binge drinkers was also significantly higher in the depressed sample, and binge drinking has been identified as an important risk factor for the development of alcohol use disorders, which has been noted in previous studies [23, 24]. Males in the clinical group reported significantly higher rates of alcohol consumption and hazardous drinking than did males in the general population. The same trend was observed for women but the differences were not significant. This might indicate that men could have a tendency to increase their alcohol intake during emotionally difficult periods more than women, as suggested previously [25]. However, due to the cross-sectional design, the relationship might be reverse. Also relevant is that depressed adults aged 28–71 years in the clinical sample exhibited more alcohol related problems than their peers in the general population. Similar differences were not found among younger adults (aged 17–27), which may indicate that older adults either are at greater risk of alcohol misuse during periods of depression, or that they are more likely to be depressed due to alcohol misuse. An alternative interpretation is that these age-related differences could reflect different response patterns among younger and older adults. The proportion of hazardous drinkers found in the depressed group is comparable to the proportion observed in other psychiatric studies in Sweden. Nehlin *et al*. [9] found that among 1811 out-patients visiting a general psychiatric clinic, 29.4 % scored above the hazardous drinking level based on the same AUDIT criteria used in the present study. Similarly, Eberhard *et al*. [26] reported that among 1670 non-psychotic outpatients, 21 % were hazardous drinkers.

Strengths of the study include the large depression sample, which offers unique insights into alcohol habits among patients in primary care. The assessment of alcohol habits, in particular hazardous drinking in outpatients with less severe depression, extends previous research focussing mainly on psychiatric inpatients [2]. We acknowledge several study limitations. In the ‘Regassa’ study it was not possible to collect information on how many individuals were invited to participate but declined, due to the administrative work required by the primary care units [13]. A second limitation was not being able to compare AUDIT data from the same years. However, consumption trends remained fairly stable between 2009 and 2013 [27] and Källmén *et al*. (2015) showed that there were insignificant changes in AUDIT scores between 2009 and 2014 [14]. The general population survey was administered during the summer months (a peak consumption period), while the clinical survey was administered throughout the year. If consumption was artificially inflated in the general survey, it is possible that differences between the study samples were underestimated. A nurse administered the AUDIT in the depressed sample but it was self-administered in the general population survey. Patients being interviewed may conceal heavy drinking habits [28] which also could reduce the magnitude of differences observed. On the other hand, underreporting may be present in the general population as well. In the clinical sample those with a *primary* substance use disorder were excluded, while there was no such exclusion in the general population. There were very few individuals with severe alcohol problems (scoring 14+/16+) in the general population, as reported by Källmén *et al*. [15] and therefore it is unlikely that including them would have a big impact on the results. What this also suggests is that the effect sizes reported here underestimate the differences in alcohol consumption between these two samples. The clinical sample is representative of people who seek treatment for common mental health problems. Depression and stress-related mental health disorders are more common among women and, as expected, the majority of participants in the clinical sample were female, while in the general population the gender distribution was more even. This may have underestimated the differences found between the clinical sample and the general population since women tend to drink less alcohol per capita.

## Conclusions

In Sweden, alcohol habits are not routinely screened in primary care settings where many patients seek help for mental health problems. Previous research has demonstrated that alcohol use can interfere with depression treatment outcomes [7, 8]. Failure to detect emerging signs of problematic drinking could increase the risk that more severe alcohol use problems will develop in the future. Our findings support the proposal that alcohol habits should be routinely screened in primary care settings when patients present with mood disorders, including milder forms of depression.
